# Utilizing Internal Hemostatic Nets for Rhytidectomy in Fitzpatrick Skin Types III to V

**DOI:** 10.1055/s-0045-1806755

**Published:** 2025-03-24

**Authors:** Shivangi Saha, Susmita Gupta, Maneesh Singhal, Vikesh Vij, Sanjay Parashar

**Affiliations:** 1 Department of Plastic, Reconstructive and Burns Surgery, All India Institute of Medical Sciences, New Delhi, India; 2 Cocoona Centre for Aesthetic Transformation and Day Surgery Hospital, Dubai, United Arab Emirates

**Keywords:** quilting sutures, platysma, SMAS, Indian skin, facelift, hyperpigmentation

## Abstract

**Introduction:**

Hematoma is a common and concerning complication following facelift surgery. To mitigate this risk, hemostatic nets can be applied either externally or internally. In patients with Fitzpatrick skin type of or greater than III, chances of dyspigmentation after external sutures are high for which internal quilting sutures (IQS) have emerged as a promising tool. There is lack of evidence on the use of IQS in darker skin types. Here, we aim to demonstrate its technique and efficacy.

**Materials and Methods:**

Forty-one individuals with Indian/Middle Eastern ethnicity, Fitzpatrick skin type ≥III underwent facelift surgery between February 2019 and October 2024. The platysma superficial musculoaponeurotic system plication facelift procedure was performed. IQS were then applied using 4–0 Vicryl, between subcutaneous tissue and skin. Patient demographic data, details of the procedure, early complications including hematoma, necrosis, bruising, nerve palsy, and late complications like skin dimpling, seroma, and sialorrhea were recorded.

**Results:**

The patient cohort comprised 6 males and 35 females (mean age: 55 years), among which 13 patients were of Mediterranean/Middle Eastern ethnicity and 28 were Asian. Fitzpatrick skin type III was present in 17 patients, type IV in 20 patients, and type V in 4 patients. The mean follow-up duration was 37.2 months. No cases of hematoma or seroma were recorded. There was one case of dehiscence in a smoker patient with diabetes. Transient dimpling was observed in seven patients, and two patients developed dog-ears; both resolved conservatively.

**Conclusion:**

In the external hemostatic net, visibility of sutures and increased risk of dyspigmentation at needle puncture sites can be distressing. IQS maintain the benefits of external hemostatic nets—such as reducing skin tension and enhancing redraping—without its disadvantages, resulting in more natural and lasting outcomes, especially for patients with thicker skin and higher Fitzpatrick skin types.

## Introduction

Hematoma is one of the most frequent and concerning complications following facelift surgery, with reported incidence rates ranging from 0.6 to 14.2%. 1 2 3 4 This complication not only increases postoperative morbidity due to ischemia, infection, fibrosis, and necrosis but also prolongs recovery by causing edema, ecchymosis, and seroma formation. 4 5 Addressing this issue has led to the development of innovative techniques aimed at reducing the occurrence of hematoma and other complications.

One such advancement is the use of quilting sutures or hemostatic nets, which can be applied either externally or internally. 6 7 8 9 These sutures work by reducing dead space between the skin and the superficial musculoaponeurotic system (SMAS), thereby significantly lowering the risk of hematoma. Beyond reducing the incidence of hematomas, hemostatic nets also minimize swelling and bruising while promoting a more uniform distribution of skin tension across the flap. This, in turn, improves blood supply, decreases ischemia and necrosis rates, and leads to better scar quality.

The use of quilting sutures offers additional benefits by allowing for more precise redraping of the skin over the SMAS, which is particularly advantageous in patients with deep wrinkles or those undergoing extensive dissection. External quilting, first introduced by Auersvald et al, has been effective, 10 but it has also been associated with complications such as hyperpigmentation at needle puncture sites, especially in patients with Fitzpatrick skin types III and IV. 6 10 Moreover, external quilting does not address the risk of delayed hematoma, leaving room for improvement in certain patient populations. 11

Internal quilting sutures (IQS) were first demonstrated by Baroudi and Ferreira in 1993, but did not gain popularity over the years. 12 Pollock and Pollock also described progressive tension suturing to shorten the convalescence and help in better tissue redraping in a small series of patients; however, this technique did not gain popularity. 8

IQS provide more robust structural support, helping reposition facial tissues while simultaneously promoting tissue regeneration and collagen production. Over time, this approach can lead to more stable, long-lasting results compared to traditional facelifts.

Despite the potential benefits, there is a paucity of literature on the use of IQS in patients with skin of color, particularly those with Fitzpatrick skin types III to V. Considering the specific characteristics of this patient population, this study aims to evaluate the technique and effectiveness of IQS (internal hemostatic nets) in higher Fitzpatrick skin types (especially Indian and Middle Eastern patients).

## Materials and Methods

This study adhered to the ethical guidelines set by the World Medical Association's Declaration of Helsinki for research involving human subjects. Between February 2019 and October 2024, 41 consecutive patients underwent facelift surgery performed by the same surgical team, and informed consent was secured from all participants. The inclusion criteria included patients with Fitzpatrick skin types III to V, Indian or Middle Eastern ethnicity, thick skin, and SMAS. These patients required face and neck lifts, with or without additional facial rejuvenation procedures, such as blepharoplasty, browpexy, and facial fat grafting. All patients were counseled regarding the possibility of transient dimpling in the early postoperative period.

Patient demographic data, including age, gender, ethnicity, smoking status, comorbidities, and Fitzpatrick skin type, were recorded. Details of each surgical procedure, including addition of submental platysmaplasty, were also documented. All patients were observed closely for early complications within the first 72 hours postsurgery, including hematoma, ischemia, necrosis, excessive bruising, swelling, and nerve palsy. Hematoma was defined as a blood collection exceeding 30 mL that required surgical drainage. 13 14 Ischemia was characterized by an area at least 1 cm ^2^ in size with a purple hue and slower capillary refill than surrounding skin. Necrosis was defined as a blackened area of at least 1 cm ^2^ with no visible perfusion. 6

Edema and bruising were graded on a scale of 1 to 5 based on patient-reported severity. 15 Preoperative and postoperative photographs were obtained. Drains were removed and patients were discharged on postoperative day (POD) 1, at which point compression garments were applied. Follow-up visits occurred on POD 7, 1 month, 3 months, and 6 months postsurgery to document delayed complications, such as skin dimpling, seroma, and sialorrhea.

## Surgical Procedure



**Video 1** Demonstration of internal quilting sutures in a facelift procedure.


The platysma-SMAS plication facelift procedure was performed under general anesthesia or intravenous sedation, combined with local anesthesia. Tumescent fluid containing 300-mL normal saline, 30-mL 2% plain lignocaine, 1.5-mL adrenaline, and 40-mg triamcinolone was injected using a 20-mL syringe with a 21-gauge needle along the incision lines, and a 1.8-mm cannula was employed to infiltrate the midface, lower face, staying above the SMAS layer and the neck and submental regions, ensuring protection of critical structures such as the marginal mandibular nerve and external jugular vein in the premasseteric area ( Video 1 ).

Next, ultrasound-/liposuction-assisted dissection was performed using a power-assisted liposuction device or suction- assisted canula, which helped bluntly dissect tissue planes in the premasseteric, lower face, submental, and neck areas. Liposuction was performed in the superficial fat compartment only, which was the plane of flap elevation for subcutaneous facelift.

Follicle-preserving incisions were made into the temporal region, followed by preauricular incisions incorporating V and Z patterns to minimize straight-line contracture. Sharp dissection proceeded deep to the subcutaneous plane, taking care to preserve the superficial temporal artery and the temporal branch of the facial nerve. The skin flap is elevated at the hypodermis level with a thin layer of subcutaneous fat. The dissection extended below the ear lobule into the neck, where the external jugular vein was carefully preserved.

The zygomatic arch was marked, and a low SMAS incision was made parallel to and 1.5 cm below the arch. A vertical SMAS incision was made in the premasseteric fascia, exposing the buccal branch of the facial nerve, which were protected throughout the procedure. The SMAS dissection was done using scissors, and hemostasis was achieved using bipolar cautery. The SMAS was then secured to the periosteum of zygoma using a three-point fixation, in a lifted position, and the buccal fat pad was suspended using 3–0 polydioxanone sutures. The jowl fat was lifted and fixed in place, while the platysma was lifted and anchored below the mandibular angle. The mandibular angle was further augmented by suspending the SMAS fat.

IQS were then applied using 4–0 rapid undyed Vicryl. These sutures were placed between the subcutaneous tissue and the SMAS at intervals of approximately 1 cm (roughly 16–20 sutures), progressively distributing the skin tension. The vector of advancement was individualized for each patient.

Redundant skin was excised, and skin flaps were trimmed and inset in a tension-free manner. The temporal skin was anchored to the superficial temporal fascia. A suction drain was placed on each side. The incisions were closed in two layers using interrupted 4–0 Vicryl and 4–0 Monocryl sutures. Absorbent dressings were placed for 24 hours and drains were removed on POD 1. IQS contributed to an addition of approximately 15 minutes to the whole duration of the facelift procedure.

## Results

Forty-one patients underwent rhytidectomy, with a mean age of 55 years (standard deviation [SD]: 8.7; range: 38–72 years). The cohort included 6 (14.6%) males and 35 (85.4%) females. Regarding ethnicity, 13 (31.7%) patients were of Mediterranean or Middle Eastern descent, while 28 were Asian. Fitzpatrick skin type III was present in 17 (41.5%) cases, type IV in 20 (48.8%) cases, and type V in 4(9.75%) cases. The mean follow-up duration was 37.2 months (SD: 13.6 months).

Of the 41 procedures, 26 (63.4%) were performed under general anesthesia and 15 (36.6%) were conducted using intravenous sedation combined with local anesthesia. The ancillary procedures performed in conjunction with rhytidectomy included 7 (17.1%) lower lid blepharoplasties, 3 (7.3%) upper lid blepharoplasties, 3 (7.3%) browpexies, 1 (2.4%) alar width reduction, and 5 (12.2%) facial fat grafting procedures.

Regarding patient comorbidities, 7 (17.1%) had diabetes mellitus, 8 (19.5%) had hypertension, 1 (2.4%) had rheumatoid arthritis, 1 (2.4%) had Sjögren's syndrome, 1 (2.4%) had glucose-6-phosphate dehydrogenase (G6PD) deficiency, 1 (2.4%) had systemic lupus erythematosus (SLE), and 1 (2.4%) had cutis laxa. Three (7.3%) patients were active smokers and three were on aspirin.

**Fig. 1:**
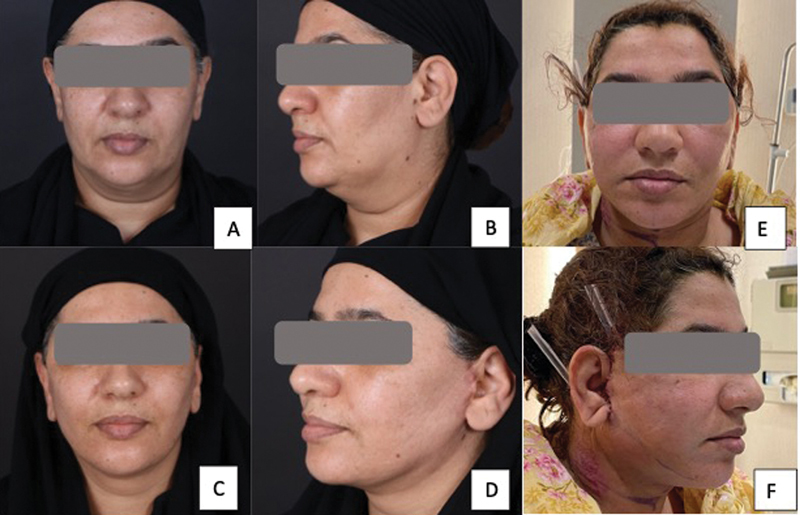
60-year-old woman following a platysma-SMAS plication facelift procedure with internal quilting sutures. ( **A, B** ) Before surgery. ( **C, D** ) After surgery. ( **E, F** ) Postoperative day 1 photographs showing minimal bruising. SMAS, superficial musculoaponeurotic system.

Postoperative complications were minimal ( Figs. 1 2 3 4 5 6 7 8 ). No cases of hematoma or seroma were recorded. There was one case of dehiscence in a patient with both diabetes mellitus and a history of smoking. Transient dimpling was observed in seven (17.1%) patients, and two (4.9%) patients developed dog-ears, both of which resolved within 3 months of follow-up.

**Fig. 2:**
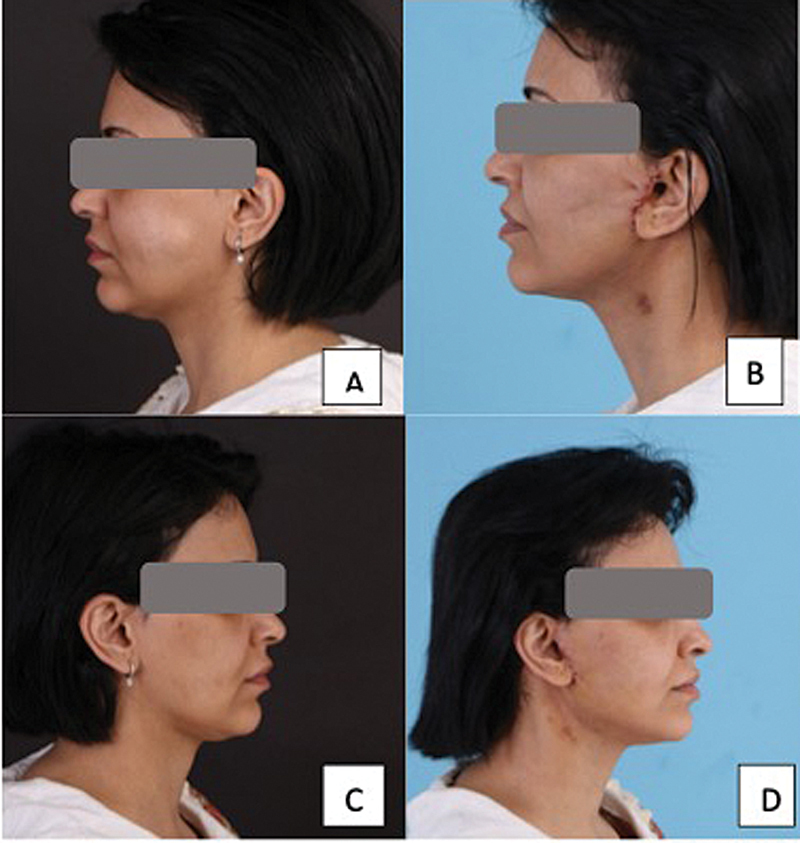
45-year-old woman following a platysma-SMAS plication facelift procedure with internal quilting sutures. ( **A, C** ) Before surgery. ( **B, D** ) Postoperative day 5 photographs showing minimal bruising. SMAS, superficial musculoaponeurotic system.

**Fig. 3:**
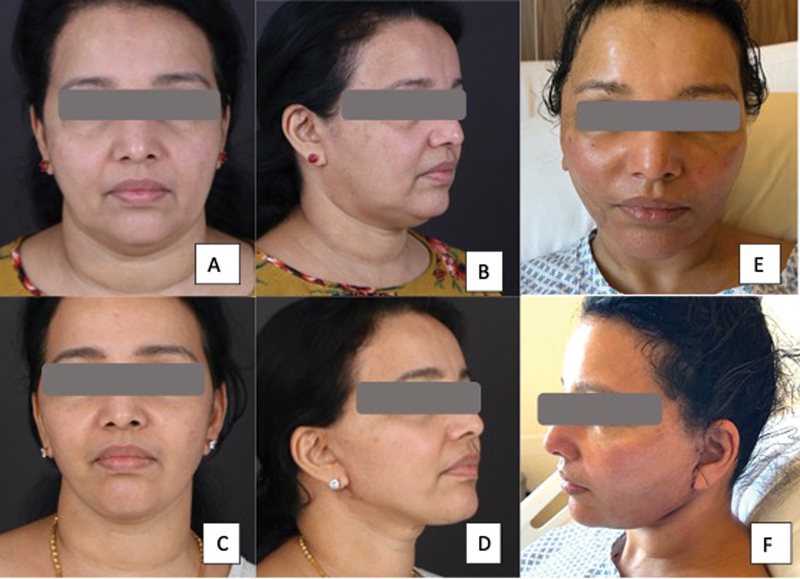
58-year-old woman a following platysma-SMAS plication facelift procedure with internal quilting sutures. ( **A, B** ) Before surgery. ( **C, D** ) After surgery. ( **E, F** ) Postoperative day 2 photographs showing minimal bruising. SMAS, superficial musculoaponeurotic system.

**Fig. 4:**
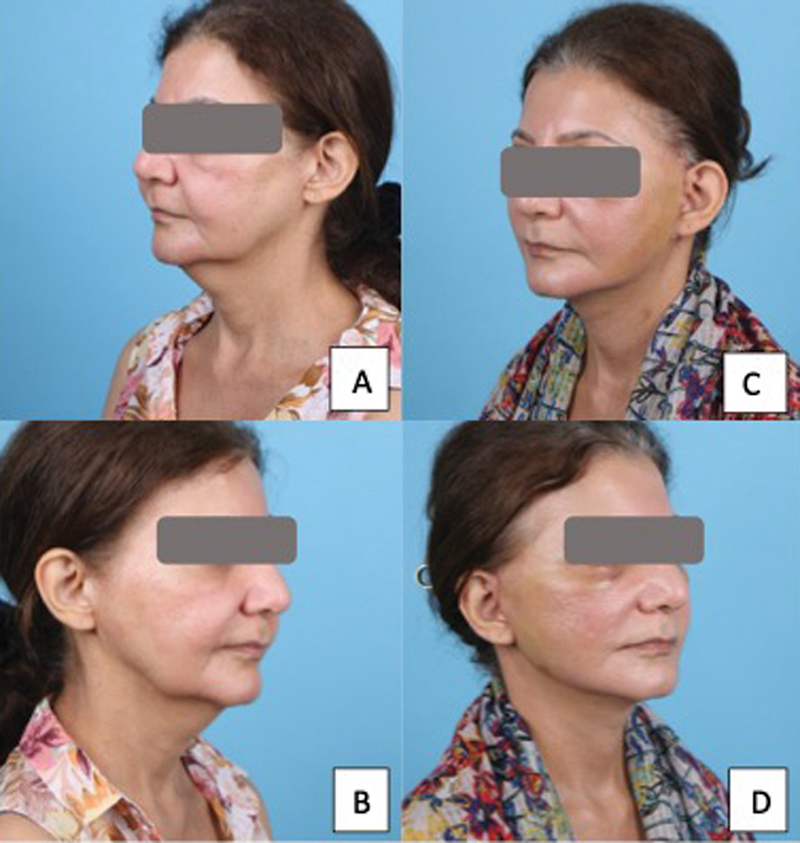
65-year-old woman following a platysma-SMAS plication facelift surgery with internal quilting sutures. ( **A, B** ) Before surgery. ( **C, D** ) Postoperative day 7 photographs showing minimal bruising. SMAS, superficial musculoaponeurotic system.

**Fig. 5:**
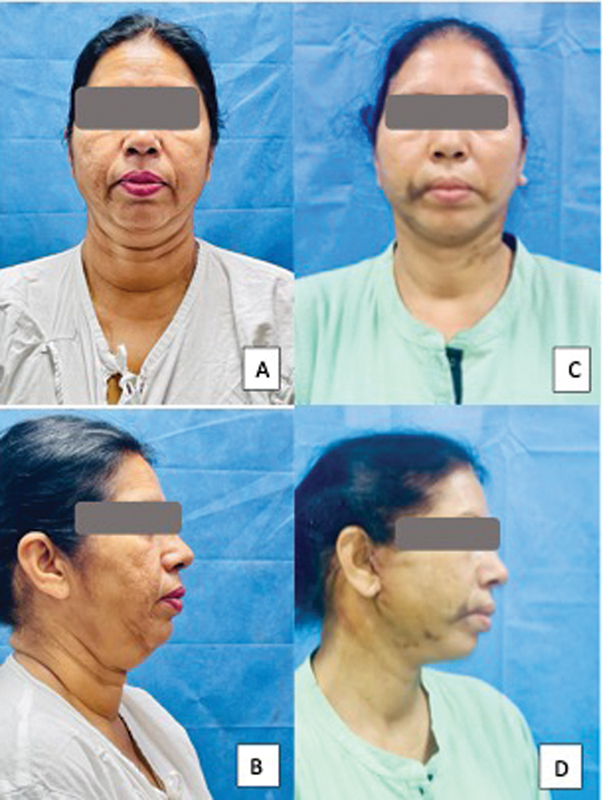
63-year-old woman following a platysma-SMAS plication facelift procedure with internal quilting sutures. ( **A, B** ) Before surgery. ( **C, D** ) Postoperative day 3 photographs showing minimal bruising. SMAS, superficial musculoaponeurotic system.

**Fig. 6:**
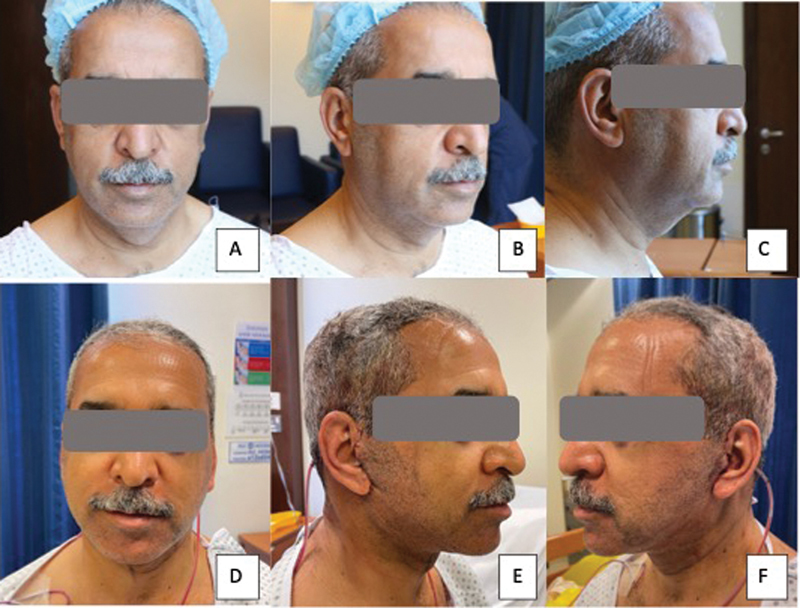
60-year-old man following a platysma-SMAS plication facelift procedure with internal quilting sutures with upper and lower lid blepharoplasty. ( **A–C** ) Before surgery. ( **D–F** ) Postoperative day 1 photographs showing minimal bruising. SMAS, superficial musculoaponeurotic system.

**Fig. 7:**
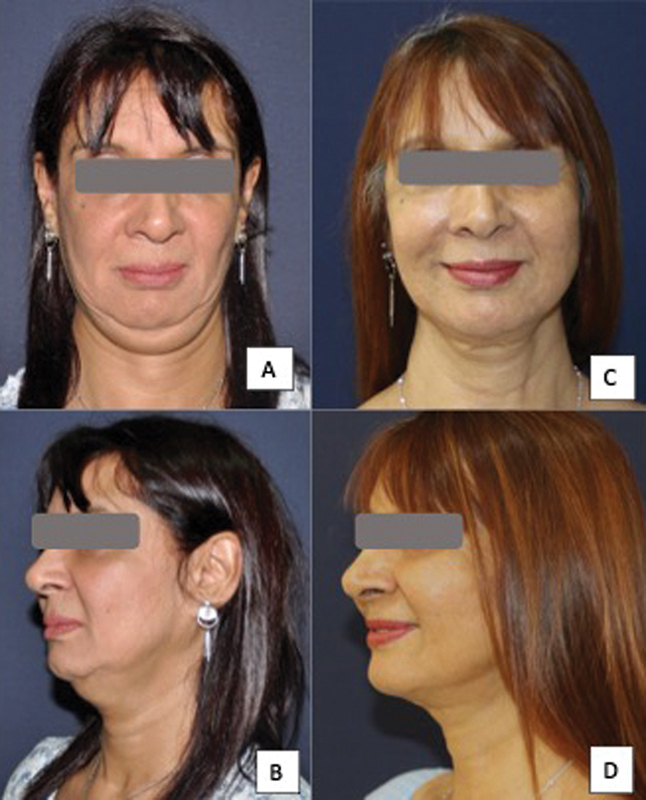
62-year-old woman following a platysma-SMAS plication facelift procedure with internal quilting sutures. ( **A, B** ) Before surgery. ( **C, D** ) Three-month postoperative photographs showing well-defined facial and neck contours. SMAS, superficial musculoaponeurotic system.

**Fig. 8:**
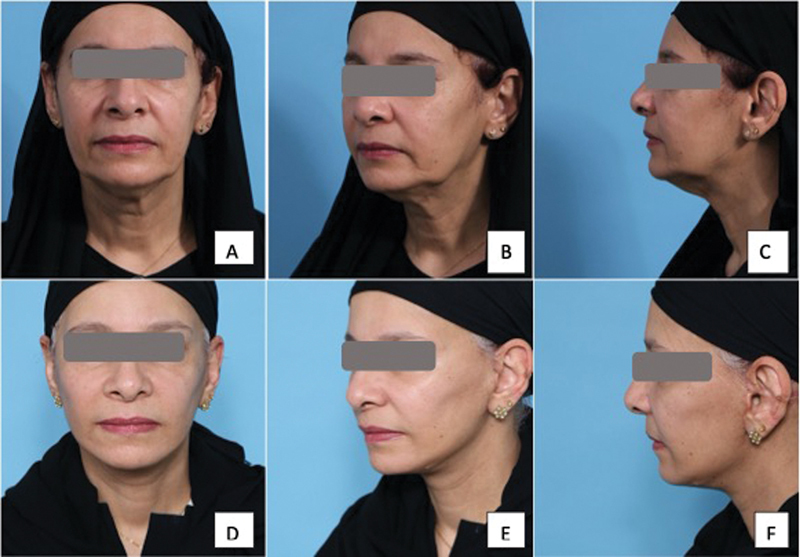
59-year-old woman following a platysma-SMAS plication facelift procedure with internal quilting sutures. ( **A–C** ) Before surgery. ( **C–E** ) Long-term postoperative results showing stable long-lasting results maintaining facial and neck contour at 6 months. SMAS, superficial musculoaponeurotic system.

## Discussion

Minimizing hematoma formation in facelift surgeries is crucial for reducing postoperative complications and surgical downtime. Hematomas not only prolong recovery but can also increase the risk of morbidity, including edema, ischemia, infection, and necrosis. Various strategies have been proposed to address this issue, including the use of fibrin sealants, multimodal blood pressure management, tumescent infiltration, exclusion of adrenaline in infiltration solutions, and low-pressure suction drains, and use of harmonic blade for dissection. 16 17 18 19 20 21 22 However, these techniques have not consistently demonstrated superiority or applicability across all patient demographics.

One established approach is the use of the Auersvald hemostatic net, 6 which has shown success in reducing hematoma incidence, though cases of delayed hematoma have been reported, particularly when sutures are removed at 48 hours. 11 To counter this, Auersvald et al extended the duration of net placement to 3 days for females and 4 days for males, providing added stability for blood coagulation in the surgical area. 23 This protocol has proven effective, especially in patients who require pharmacological anticoagulation, without posing additional risks to healing. This extended timeline is pertinent especially for patients on thromboembolism prophylaxis, as it helps further mitigate hematoma risk.

Auersvald et al have recently advocated for the use of a 5–0 nylon suture with a 30-mm 3/8 cutting needle (Monosof; Covidien, Minas Gerais, Brazil), which they found particularly beneficial for patients with thicker skin and more substantial subcutaneous tissue, such as those with Indian skin types. However, availability of this specific needle is limited globally. 23 In the instances where the preferred needle is unavailable, our proposed approach of using internal suturing between the skin and SMAS can still be achieved with smaller needles, potentially without compromising efficacy.

External hemostatic sutures, while effective, bring added challenges, including an initial “ghastly” appearance that can be unsettling for patients. Additionally, leaving external sutures in place for 4 days may increase the risk of hyperpigmentation, especially in patients with Fitzpatrick skin types III to V. Notably, there are no published data on the impact of the Auersvald modified protocol on patients of color, an area that warrants further exploration to ensure safe and aesthetic outcomes in diverse populations. 22 In a previous study by Auersvald et al, 17.1% of patients, particularly those with darker skin types (Fitzpatrick III and IV), experienced hyperpigmentation at needle puncture sites, while three patients developed persistent hypopigmentation. 6 10 To manage this, they employed topical hydroquinone 2% for a month.

There are limited data on the long-term effects of this approach, especially in patients of color. Moreover, the removal of external sutures afterward often requires the expertise of the surgeon or well-trained staff, adding to the postoperative burden. There is still a risk of seroma formation following suture removal, which can complicate recovery.

Risk of hematoma under SMAS is a very limited risk and needs direct visualization and coagulation using bipolar cautery. Subcutaneous hematoma is a bigger risk as there is significant degloving of skin and small subdermal vessels can bleed postoperatively.

The role of drains alongside hemostatic sutures in facelifts is still debated. Although internal hemostatic sutures can significantly reduce dead space and mitigate fluid accumulation, drains may still serve as a useful adjunct by providing a conduit for residual fluid efflux, particularly in cases with extensive tissue manipulation or patients at higher risk of fluid retention. 24 Studies examining the use of quilting sutures in abdominoplasty, for instance, have found that drains remain beneficial in reducing postoperative seroma, suggesting a potential parallel benefit in rhytidectomy. 9 25

In contrast, IQS, or an internal hemostatic net, offer several advantages over the external method ( Table 1 ). Internal quilting preserves the benefits of external hemostatic nets, such as reducing skin tension and enhancing skin redraping, without the need for visible sutures. The possibility of hypertrophy of scar, stretching of scars and pixie ears are more if tension is directly on the skin or subcutaneous sutures. By progressively reducing tension on the skin flaps, internal sutures facilitate more precise tissue repositioning, leading to a more natural and aesthetically pleasing result. Additionally, the internal technique eliminates the need for suture removal and avoids the appearance-related concerns and hyperpigmentation risks associated with external quilting.

**Table 1:** Summary of the advantages of Internal Quilting Sutures

1	Provide natural results	The IQS technique provides superior tissue repositioning, resulting in less tension on the suture line and better skin redraping
2	Quick downtime	It offers effective hemostasis and, thus, can shorten recovery time by reducing the edema and excessive bruising compared to traditional facelift techniques
3	Minimizing complications	By reducing dead space and providing better support for facial tissues, the internal hemostatic net decreases the likelihood of complications such as hematoma, seroma, and prolonged swelling
4	Long-term effectiveness	The structural support offered by internal quilting sutures contributes to more stable and long-lasting results, maintaining facial contours and minimizing skin laxity over time

The only contraindication was when the skin was very thin, but in our series of patients with skin types III, IV, and V, the skin was found to be thick enough to place internal sutures without risks. As the skin flap was elevated at the hypodermis level with a thin layer of subcutaneous fat, there was occasional risk of dimpling, but it was found to be transient, as 4–0 Vicryl Rapid dissolves within 2 weeks. Transient dimpling was observed in seven (17.1%) patients, which resolved within 3 months of follow-up.

We did not objectively measure skin flap thickness in our case series. However, a prospective study that quantifies the exact thickness of the skin flap and fat layer would provide a more systematic approach to determining whether external or internal quilting is better suited for different skin and subcutaneous tissue thicknesses.

## Conclusion

While external quilting sutures have provided valuable improvements in facelift surgeries, the IQS/hemostatic net (IQS) technique offers a refined, less invasive alternative. It not only reduces complications and improves recovery but also achieves more natural, enduring results, especially in patients with thicker skin and higher Fitzpatrick skin types.
